# “The Real Cost” Smokeless campaign: changes in beliefs about smokeless tobacco among rural boys, a longitudinal randomized controlled field trial

**DOI:** 10.1186/s12889-021-12356-6

**Published:** 2021-12-14

**Authors:** Matthew C. Farrelly, Nathaniel H. Taylor, James M. Nonnemaker, Alexandria A. Smith, Janine C. Delahanty, Xiaoquan Zhao

**Affiliations:** 1 Center for Health Analytics, Media, and Policy, RTI International, 3040 E. Cornwallis Road, Research Triangle Park, NC 27709 USA; 2 Center for Tobacco Products, U.S. Food and Drug Administration, Silver Spring, MD USA; 3 Department of Communication, George Mason University, Fairfax, VA USA

**Keywords:** Smokeless tobacco, Rural youth, Media campaign evaluation, Longitudinal, Randomized

## Abstract

**Background:**

The prevalence of current smokeless tobacco (SLT) use in 2019 among high school students was 4.8%, and the overall rate of SLT use was higher among high school boys (7.5%) than girls (1.8%). The U.S. Food and Drug Administration (FDA) launched “The Real Cost” Smokeless media campaign in April 2016 to educate rural youth about the dangers of SLT use. In this study, we evaluate the effectiveness of “The Real Cost” Smokeless campaign.

**Methods:**

We use a 3-year (Jan 2016 – Dec 2018) randomized controlled longitudinal field trial that consists of a baseline survey of boys and a parent/guardian and four follow-up surveys of the boys. The cohort includes 2200 boys who were 11 to 16 years old at baseline and lived in the rural segments of 30 media markets (15 treatment markets and 15 control). **“**The Real Cost” Smokeless campaign targets boys who are 12 to 17 years old in 35 media markets. It focuses primarily on graphic depictions of cosmetic and long-term health consequences of SLT use. The key outcome measures include beliefs and attitudes toward SLT that are targeted (explicitly or implicitly) by campaign messages.

**Results:**

Using multivariate difference-in-difference analysis (conducted in 2019 and 2020), we found that agreement with 4 of the 11 explicit campaign-targeted belief and attitude measures increased significantly from baseline to post-campaign launch among boys 14 to 16 years old in treatment vs. control markets. Agreement did not increase for boys 11–13 years old in treatment vs. control markets and only increased for one targeted message for the overall sample.

**Conclusions:**

These findings suggest that “The Real Cost” Smokeless campaign influenced beliefs and attitudes among older boys in campaign markets and that a campaign focused on health consequences of tobacco use can be targeted to rural boys, influence beliefs about SLT use, and potentially prevent SLT use.

**Supplementary Information:**

The online version contains supplementary material available at 10.1186/s12889-021-12356-6.

## Background

The prevalence of current smokeless tobacco (SLT) use in 2019 among high school students was 4.8%—similar to the prevalence of cigarette smoking (5.8%) and lower than cigar use (7.6%) [1]. However, the overall rate of SLT use masks significant differences by gender and geography. In 2019, 7.5% of high school boys used SLT compared with 1.8% of girls [1]. Wiggins and colleagues found that high school students in rural areas were twice as likely to use SLT as students in urban areas, controlling for gender, race/ethnicity and survey year in an analysis of the National Youth Tobacco Survey from 2011 to 2016 [2]. These patterns are similar among adults, with current use in rural areas at 8.1% compared with 2.5% in urban areas [3]. Among adults, the gender differences are even more pronounced, with current SLT use prevalence of 5.7% for men and 0.2% for women [3]. Although some argue that SLT use is a safer alternative to cigarette smoking, SLT use is not harmless. Its use causes oral, esophageal, and pancreatic cancer and oral mucosal lesions, leukoplakia, and periodontal disease [4].

To educate youth about the dangers of SLT use, the U.S. Food and Drug Administration (FDA) expanded “The Real Cost” campaign in April 2016 to target rural male youth at risk of or already experimenting with SLT use. The campaign messages, built on extensive qualitative research and theories of health behavior change [5], focused largely on graphic depictions of cosmetic and long-term health consequences of SLT use [6]. The campaign identified knowledge gaps among the target audience such as progression of consequences from white spots to gum disease and pre-cancerous tumors. Campaign messaging also emphasized that safer (than cigarettes) does not mean safe and nicotine addiction can lead to a loss of control. The campaign used broadcast television, digital video (e.g., YouTube), radio, and social media to reach rural boys with campaign messages. The FDA media contractor purchased advertisements (ads) in 35 Designated Market Areas (DMAs) that were comprised of rural counties with a high prevalence of SLT use. Our control markets included similar counties (i.e., rural, high SLT use) that were not included in the media buy. To our knowledge, “The Real Cost” Smokeless campaign is the largest SLT prevention campaign in the U.S. and the only large-scale effort since the National Spit Tobacco Education Program by the National Cancer Institute and Robert Wood Johnson Foundation in the late 1990s [7].

To evaluate the effectiveness of “The Real Cost” Smokeless campaign, we conducted a randomized controlled longitudinal field trial with proportional allocation that consisted of a baseline survey of boys and a parent/guardian and four follow-up surveys of the boys. The boys were 11 to 16 at baseline and lived in the rural segments of 30 media markets. This manuscript describes the findings from this 3-year longitudinal study.

## Methods

### Study design and data collection

To identify DMAs for the study, FDA developed a list of the 47 markets with the largest populations of youth who were at risk of or experimenting with SLT. To ensure that the campaign could reach a sufficiently large population nationwide, we excluded the 12 most populous markets from the randomization. We then randomly selected 15 intervention (markets received campaign ads) and 15 control (did not receive campaign ads) markets for the longitudinal study from the next most populous 30 markets, which served as the primary sampling units. Although respondents in control markets were not exposed to campaign ads, all respondents were shown the campaign’s video ads in the survey to assess campaign awareness. The campaign targeted rural segments (defined as C and D Nielsen counties) of the intervention DMAs. To ensure we would have sufficient sample to detect the influence of the campaign on campaign-targeted beliefs, we conducted a power calculation that indicated we would need 1008 youth by the final wave of data collection. Once we factored in anticipated longitudinal retention, our goal was to complete 1969 baseline surveys. However, a sampling error at baseline led to the inclusion of a small fraction of households that were more suburban than rural. As a result, we increased the baseline sample to 2200, including 1895 from rural counties. We used address-based sampling, drawing household addresses from Census Block Groups (the secondary sampling units) in the 30 markets. The groups were allocated proportionally to the size of the DMAs. We selected the address samples from the Census Block Groups and used the number of boys aged 11 to 16 years old as the size measure. Our third stage sampling units were addresses from the Computerized Delivery Sequence file. We sampled approximately 100 addresses per selected Census Block Group.

In January 2016, we sent paper and pencil household screeners with a $2 pre-paid incentive to identify households with potentially eligible boys 11 to 16 years old (allowing more than one boy per household). We then sent field interviewers to households to conduct in-person baseline surveys with youth and parent/guardians.

Field interviewers obtained parental permission and youth assent, provided youth with instructions on how to complete the interview on a laptop, and were available to answer questions during the self-administered survey. Youth who completed the baseline survey received a $20 cash incentive. Once each youth respondent started taking the survey, field interviewers provided the parent/guardian with instructions on how to complete the parent/guardian survey on a tablet. Parents were not offered an incentive for the baseline survey.

We followed up with youth every 8 months from the start of the previous wave with the final wave of data collection ending in December 2018. At each follow-up, we contacted parents of youth (and youth directly who were at least 18 years old) by mail and email and invited them to provide permission for their child to complete the survey on the web. For youth who completed the survey within the first 4 weeks, we offered an additional $5 “early-bird” incentive (total of $25 by check). Field interviewers contacted youth who did not respond to the web survey during the early-bird period and reminded them to complete the web survey or scheduled an in-person interview if youth were not able to complete the survey online. The incentive after the early-bird period was a $20 check if they completed the survey online or $20 in cash if they completed the survey in-person.

At each wave of data collection, we monitored responses for quality and removed responses that did not meet our quality standards. We reviewed the data to ensure that respondents did not speed through the survey (complete a survey in less than 5% of the mean time), fail both attention check questions, and straight-line (i.e., choose the same answer in a column of questions) more than 66% of possible items that could be straight-lined. Respondents who failed our quality controls received an incentive but were not invited to subsequent waves of data collection.

### Measures

The key outcome measures include beliefs and attitudes toward SLT that were targeted (explicitly or implicitly) by campaign messages. We analyzed 25 beliefs and attitudes to determine if they were related, or unrelated, to the campaign (list of beliefs shown in Supplement 1). Three coders reviewed “The Real Cost” Smokeless ads to identify belief items that explicitly targeted, implicitly targeted, or were not related to the campaign messages. Rater agreement was high (overall: Gwet’s AC [8] = 0.84; ads ranged from 0.65 to 0.91). We found that 12 beliefs were messaged explicitly in “The Real Cost” Smokeless, 6 were covered implicitly, and 7 beliefs that we presented to respondents were unrelated to the campaign messages. As there were no statistically significant changes in the latter two categories, we focus the results on the explicit messages. However, we do not present results from one of the twelve campaign-targeted beliefs (Lose my jaw) because we did not collect data for that belief until third follow-up. The beliefs corresponding to these explicit messages related to nicotine dependence (e.g., unable to stop when I want to), short-term health effects (e.g., Develop red or white patches in the mouth), long-term health effects (e.g., Develop cancer of the lip, mouth, tongue, or throat), and social influences (e.g., Miss out on things I enjoy doing). Implicit beliefs included social influences (e.g., fit in) and perceived risk (e.g., safe to use SLT for a year or two). Unrelated beliefs included health consequences (e.g., get sick more often) and perceived benefits (e.g., using SLT relieves stress). Study participants indicated their agreement with belief and attitude statements on a 5-point Likert scale from strongly agree to strongly disagree. For analysis we dichotomized these items as strongly agree/agree (1) vs. other responses (0). The key intervention variable is a dichotomous variable for being in a treatment DMA (1) or in a control DMA (0).

The constructs from the baseline survey of parents/guardians that we use in the analysis include race (White, non-Hispanic, all other races/ethnicities (referent)), education (less than high school/high school diploma or more (referent)), and household income (less than $30,000, $30,000–$49,999, $50,000–$69,999, $70,000 or more). We also asked parent/guardians about their employment status (employed/unemployed (referent)), marital status (married/not married (referent)), and about their relationship to the child (biological parent/else (referent)). We asked youth about their sensation seeking behaviors at baseline. To measure this construct, we created a composite dichotomized scale derived from a 5-point Likert scale (strongly agree to strongly disagree) of the following: explore strange places, do frightening things, break the rules to do new and exciting things, and prefer friends who are exciting and unpredictable [9]. We took the average response among those four items and dichotomized those averages at the mean, where sensation seekers included the mean response and above, for analysis [10].

In addition to baseline sensation seeking behavior, our analyses include several youth measures asked at each wave of data collection and therefore responses varied over time. Measures include media use/exposure: how often they watched TV at least once a day vs. less than once a day (referent); used any one of four social media platforms at least once a day including Facebook, Instagram, Twitter, or Snapchat vs. less often (referent); used any one of five streaming services at least once a day including YouTube, Twitch, Netflix, Hulu, or Amazon Prime vs. less (referent); played video games (at least once a day vs. less often (referent)); frequency of watching R-rated movies (sometimes or more vs. less); parents have lots of rules about computer use, video games, and type of music vs. few or no rules (referent); their awareness of the truth tobacco prevention campaign (aware vs. not aware (referent)); and a fake tobacco prevention campaign (aware vs. not aware (referent)) to account for false reporting.

Other youth variables include use of SLT and cigarettes by family members in past 30 days vs. no use in past 30 days (referent), school performance (much better than average, better than average, average or below (referent)), and church attendance (at least once a week vs. less often (referent). We measured school environment using an average of three measures: feeling close to people at school, happy to be at school, and feeling like they were a part of their school. We divided those averages into tertiles using the bottom tertile as the referent. Finally, we captured the influence of their friends with two separate measures: “I do what my friends want me to do, even if I don’t want to” (Strongly agree/agree vs. else) and “To keep my friends, I’d even do things I don’t want to do” (strongly agree/agree vs. else).

### Analytic methods

We analyzed data between 2019 and 2020 starting with an examination of changes in agreement (strongly agree and agree) with campaign-targeted beliefs and attitudes from baseline to each follow-up for the treatment and control groups. We reverse coded variables that contained affirmative beliefs (e.g., If I use smokeless tobacco, I will fit in) such that disagreement (strongly disagree and disagree) was coded as 1 to make comparisons with negative messages easier to interpret. We then calculated the difference pre-campaign to post in agreement for the treatment group (Tx), the difference pre to post in agreement for the control group (Cx), and the difference of these differences (DID).

$$\left(\mathrm{Tx}\ {\mathrm{Agreement}}_{\mathrm{Post}}-\mathrm{Tx}\ {\mathrm{Agreement}}_{\mathrm{Baseline}}\right)-\left(\mathrm{Cx}\ {\mathrm{Agreement}}_{\mathrm{Post}}-\mathrm{Cx}\ {\mathrm{Agreement}}_{\mathrm{Baseline}}\right)$$

DID isolates the changes over time that are associated with the campaign. We did this for the overall sample and stratified by age (11–13, 14–16 at baseline) to test if boys of different ages reacted differently to the campaign. To examine early campaign results, we also report changes from the baseline to the second follow-up. We conducted multivariate DID models with a treatment group indicator, pre-post campaign indicator, and the interaction between the two indicators (i.e., treatment*pre-post) with the full sample and stratified by age. We then used margins [11] to estimate the DID in percentage point terms for each outcome variable. Our models include control variables described above and dropped 81 observations for missing responses in the multivariate models vs. models without control variables. Finally, we tested if being in the treatment group, along with model covariates, was associated with attrition by creating an indicator variable for respondents who dropped out after any wave of data collection and did not return to complete a subsequent survey.

## Results

### Sample characteristics

**Fig. 1:**
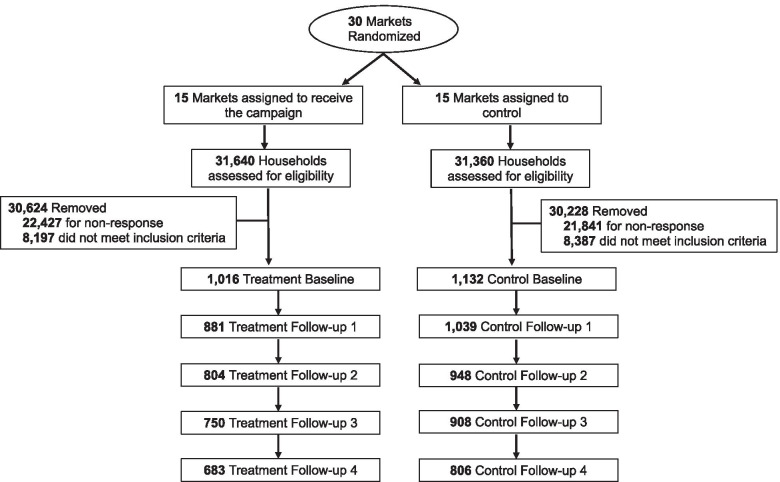
Consort Diagram

We sent 63,000 screeners yielding 18,734 (nearly 30%) completed screeners and 2885 potentially eligible households. Field interviewers successfully completed baseline surveys with youth in 76.3% (*N* = 2200; treatment = 1058; control = 1142) of the eligible households and 1827 parent/guardians in those households. Longitudinal retention rates, excluding those ineligible or unable to participate were 92% at follow-ups 1 and 2 and 89% at follow-ups 3 and 4. The follow-up sample sizes were 1937, 1770, 1667, and 1490. We withdrew 252 participants for the following reasons: moved out of the study area (*n* = 173), became ineligible because of age (*n* = 15), someone other than the enrolled participant completed a survey (*n* = 35), poor data quality (*n* = 20), institutionalized (*n* = 5), and deceased (*n* = 4). (Fig. 1).

The sample was mostly White, non-Hispanic (81.2%) but also included 7.0% Hispanic, 3.2% Black, non-Hispanic, and 8.6% other race/ethnicities or multiracial. At baseline, the sample was evenly split between boys who were 11–13 years old (49.2%) and 14–16 (50.8%). Most of the sample lived in rural communities (88.2%) and 7.8% had ever tried SLT. Additionally, 12.9% lived in households with a family member who used SLT in the past month at baseline. There were significantly more White, non-Hispanic youth in the treatment group (84.6%) than in the control group (80.3%) at baseline (*p* < 0.01) and less Hispanic youth in treatment (5.1%) than in the control (8.9%; *p* < 0.001). There was more SLT use in the treatment group (4.4%) than in the control group (2.2%) at baseline (*p* < 0.01) and generally more other tobacco product use in treatment than in the control (Table 1). We did not receive any reports of unintended effects or harms. We analyzed data from 1016 respondents in the treatment group and 1132 in the control group for the main outcomes after removing ineligible responses. Once assigned to the treatment or control group, respondents did not move between groups. The attrition analysis found that although being in the treatment condition was not associated with attrition, dropping out of the study was associated with several baseline measures—using SLT, being in the older age group, living in a rented home, parents having lots of media use rules, and youth reporting awareness of a fake tobacco campaign. Having a biological parent complete the parent/guardian survey and the interviewer assessing the parent/guardian as cooperative during the interview were both protective against attrition.

**Table 1:** Baseline Demographics and Tobacco Use

Demographics	Treatment (***n*** = 1016)	Control (***n*** = 1132)	***P*** Value
**Age**			
11–13	49.9%	48.7%	0.587
14–16	50.1%	51.3%	0.587
**Race/Ethnicity**			
White, NH	84.6%	80.3%	**0.008**
Black, NH	3.8%	2.7%	0.169
Hispanic	5.1%	8.9%	**0.000**
American Indian/Alaska Native	0.9%	1.3%	0.409
Asian	0.4%	0.8%	0.215
Multiracial/Other	5.3%	6.0%	0.488
**Ever Smokeless Tobacco Use**	9.4%	6.3%	**0.007**
**Current Tobacco Use (Past 30 Days)**			
Household SLT use	15.5%	10.6%	**0.000**
Smokeless tobacco	4.4%	2.2%	**0.004**
Cigarettes	2.8%	1.5%	**0.046**
Cigars, cigarillos, or little cigars	2.8%	1.6%	0.067
Hookah	0.3%	0.4%	0.813
Vape	5.5%	3.7%	**0.048**
**Nielsen County Rank**			
B	8.1%	14.8%	**0.000**
C	22.5%	34.6%	**0.000**
D	69.0%	50.6%	**0.000**

### Beliefs and attitudes

In unadjusted DID analyses, we found that agreement with 3 of the 11 explicit campaign-targeted belief and attitude measures increased significantly from baseline to post-campaign launch (all follow-up waves included) in the treatment group compared with control, measured in percentage point changes (Table 2). Those belief and attitude measures were: If I use smokeless tobacco, I will … [1] damage my body (+ 3.5; *p* < 0.05) [2]; be unable to stop when I want to (+ 7.5; *p* < 0.01); and [3] develop gum disease (+ 3.7; *p* < 0.05). Agreement did not change significantly for implicit campaign-targeted or unrelated beliefs and attitudes. We stratified the sample by age and found no significant changes in agreement from baseline to post-campaign launch with any beliefs or attitudes (campaign-targeted and unrelated) for the younger age group. In the older age group, agreement increased significantly from baseline to post-campaign launch for five beliefs, including all three of the beliefs mentioned above, as well as “If I use smokeless tobacco, I will shorten my life” (+ 5.6; *p* < 0.05) and “Be controlled by smokeless tobacco” (+ 7.9; *p* < 0.05). When we analyzed changes from baseline to second follow-up, we found significant increases in agreement with the same measures as the overall pre-post analysis and additionally “I will be controlled by smokeless tobacco” (+ 5.0; *p* < 0.05). In addition, the magnitude of changes was larger for this shorter period than for the full study. A closer examination of agreement with campaign-targeted belief and attitude measures among the overall sample and by age group (not shown) suggests that the increases in agreement in the treatment group reached a plateau at second follow-up for both age groups, whereas agreement in the control group continued to increase over time.

**Table 2:** Unadjusted Difference-in-Difference

Belief	DID All Waves (***n*** = 8950)	DID All Waves (Ages 11–13) (***n*** = 4442)	DID All Waves (Ages 14–16) (***n*** = 4437)	DID Baseline to Follow-up 2 (***n*** = 5807)
**If I use smokeless tobacco, I will**				
Damage my body	3.5*	0.8	6.2**	4.8**
Be controlled by SLT	4.2	0.7	7.9*	5.0*
Develop cancer of the lip, mouth, tongue, or throat	1.2	0.4	2.0	2.6
Be unable to stop when I want to	7.5**	4.0	10.9**	8.7***
Lose my teeth	2.4	0.5	4.3	3.4
Shorten my life	2.7	−0.3	5.6*	3.4
Miss out on things I enjoy doing	2.5	0.9	4.3	3.4
Develop gum disease	3.7*	0.3	7.1**	4.7*
Develop red or white patches in the mouth	1.4	−2.2	5.1	2.5
Consume harmful chemicals	1.5	0.7	2.3	2.5
Cause immediate damage to my body	−3.2	−4.5	−1.9	−1.5

^a^All variables are coded: 1 = Strongly Agree/Agree; 0 = Neutral/Disagree/Strongly Disagree

^b^Cells present unadjusted percentage point changes (Δ Treatment – Δ Control)

^c^Boldface indicates statistical significance (* *p* ≤ 0.05, ** *p* ≤ 0.01, *** *p* ≤ 0.001)

The multivariate analyses confirmed the unadjusted model changes for older boys except for “shorten my life.” Changes in agreement among the younger age group remained insignificant. In the overall sample, only “Be unable to stop when I want to” was significantly associated with “The Real Cost” Smokeless campaign in the full study period (Table 3). However, through second follow-up, changes in agreement with “Be unable to stop when I want to” and “Damage my body” were associated with being in the treatment group.

**Table 3:** Multivariate Difference in Difference

Belief	DID All Waves (***n*** = 8869)	DID All Waves (Ages 11–13) (***n*** = 4442)	DID All Waves (Ages 14–16) (***n*** = 4411)	DID Baseline to Follow-up 2 (***n*** = 5753)
**If I use smokeless tobacco, I will**				
Damage my body	2.7	0.5	4.8*	4.0*
Be controlled by SLT	3.8	0.8	6.8*	4.4
Develop cancer of the lip, mouth, tongue, or throat	1.0	0.7	1.1	2.1
Be unable to stop when I want to	7.1**	4.3	10.1**	8.1**
Lose my teeth	1.9	0.9	3.1	2.8
Shorten my life	2.2	−0.2	4.5	2.9
Miss out on things I enjoy doing	1.5	0.9	2.3	2.2
Develop gum disease	3.1	0.7	5.6*	3.8
Develop red or white patches in the mouth	0.6	−2.5	3.5	1.5
Consume harmful chemicals	0.9	0.7	1.1	1.4
Cause immediate damage to my body	−4.2	−5.2	−3.2	−2.6

^a^All variables are coded: 1 = Strongly Agree/Agree; 0 = Neutral/Disagree/Strongly Disagree

^b^Cells present adjusted percentage point changes (Δ Treatment – Δ Control)

^c^Boldface indicates statistical significance (* *p* ≤ 0.05, ** *p* ≤ 0.01, *** *p* ≤ 0.001)

^d^Multivariate models controlled for the youth’s age and race, parent’s education, household income, work status, and marital status, biological parent vs. not, youth’s use of TV, social media, streaming services, and video games, youth awareness of the truth campaign and a fake media campaign (for an additional control), youth sensation seeking, school performance and church attendance, youth willingness to do things they don’t want to do in order to keep friends or please friends, use of SLT or cigarettes by others in the household, rules about media use and watching R-rated movies, and whether the youth completed the survey in person or online

## Discussion

In early 2016, FDA supported a 3-year, randomized controlled longitudinal field trial that delivered messages with graphic depictions of the health effects, addictive nature, and social consequences of SLT use with broadcast and digital media strategies to reach boys 12 to 17 living in rural segments of 15 media markets (with 15 control markets). After approximately 15 months, descriptive and multivariate results of this rigorous study design indicate that “The Real Cost” Smokeless influenced several campaign-targeted beliefs and attitudes related to addiction and health consequences in the overall sample. The timing of these changes is consistent with other tobacco prevention campaigns [12–15]. Changes in beliefs in the treatment group reached a plateau in subsequent follow-ups, while agreement with beliefs in the control group increased steadily and modestly. As a result, the initial impact appeared to fade by the end of the study across all ages. The steady level of agreement in campaign-targeted beliefs in the treatment group after follow-up 2 may be explained by the fact that there were only two new video ads introduced after follow-up 2—one that aired prior to and during follow-up 3 and one concurrent with follow-up 4.

However, when we examined trends by age group, we found changes in beliefs related to addiction and health consequences for boys ages 14 to 16 at baseline that remained statistically significant through final follow-up. In contrast, the campaign did not influence boys 11 to 13 at baseline in the short- or long-run. Given that the average age of SLT use initiation is nearly 14 years old [16], younger boys may have found the campaign less salient to them than the older boys. In addition, beliefs pertaining to social consequences of SLT use (e.g., Miss out on things I enjoy doing) did not change for any age group.

Two additional beliefs targeted by explicit campaign messages, “develop red or white patches in the mouth” and “develop cancer of the lip, mouth, tongue, or throat” also did not change for either age group. The message of developing red or white patches in the mouth was mentioned in an early campaign ad but was not consistently featured until third follow-up and therefore may not have had enough time to influence beliefs. Develop cancer was the focus of several ads throughout the campaign but agreement with this belief was high at baseline (84% overall agreement), which did not allow room for significant growth.

The campaign focused on health consequences of tobacco use and results from this evaluation provide additional evidence that public education campaigns with clearly stated and graphic messages about health consequences are effective. FDA’s flagship general audience smoking prevention campaign—“The Real Cost”—used a similar strategy and was effective in influencing campaign-targeted beliefs and smoking behavior [12, 17]. Although this study was not designed to have sufficient statistical power to detect changes in behavior, changes in beliefs and attitudes have been shown to be predictive of downstream behavior change [17–19].

Although the current study followed a rigorous design, it is not without limitations. All study participants were shown the campaign’s video ads at each follow-up survey to assess reach. Campaign implementation data suggests minimal delivery of campaign messages in the control markets and self-reported awareness of any video ad in control markets averaged 44.5% in follow-up surveys compared to 82.0% in treatment markets, but it is possible that this minimal exposure was enough to have a modest change in beliefs over time and helps explain some of the convergence between the treatment and control groups.

Although attrition at each longitudinal wave was limited to approximately 10%, by the end of the study, 32% of the baseline sample was lost to follow-up. In addition, although there was no statistically significant association between attrition and the treatment condition—that is, no systematic attrition by treatment status—this does not rule out bias. Further, SLT use at baseline was associated with attrition at any wave of data collection and was statistically different between treatment and control groups. However, we did not control for baseline SLT use because of concerns about endogeneity and the simultaneous effect that SLT use has on belief outcomes and vice versa.

## Conclusions

Despite these potential limitations, the totality of the findings suggest that “The Real Cost” Smokeless campaign influenced beliefs and attitudes among older boys in campaign markets. These finding suggest that a campaign focused on health consequences of tobacco use can be targeted to rural boys, influence beliefs about SLT use, and potentially prevent SLT use.

## Supplementary Information

**Additional file 1.**

## Data Availability

The datasets generated and/or analysed during the current study are not publicly available due to privacy concerns but are available from the corresponding author on reasonable request.

## Abbreviations

SLT: Smokeless tobacco
FDA: U.S. Food and Drug Administration
DMA: Designated market area
DID: Difference-in-difference

## References

[1] Wang TW, Gentzke AS, Creamer MR (2019). Tobacco product use and associated factors among middle and high school students—United States, 2019. [accessed April 30, 2020]. MMWR Surveill Summ.

[2] Wiggins AT, Huntington-Moskos L, Rayens EA (2020). Tobacco use among rural and urban US middle and high school students: National Youth Tobacco Survey, 2011–2016. J Rural Health.

[3] Cheng YC, Rostron BL, Day HR (2017). Patterns of use of smokeless tobacco in US adults, 2013–2014. Am J Public Health.

[4] National Cancer Institute and Centers for Disease Control and Prevention (2014). Smokeless tobacco and public health: a global perspective.

[5] Fishbein M, Azjen I (1975). Belief, attitude, intention, and behavior: an introduction to theory and research.

[6] Walker MW, Evans SA, Wimpy C (2018). Developing smokeless tobacco prevention messaging for at-risk youth: early lessons from “the real cost” smokeless campaign. Health Equity.

[7] Koppett L (2000). The National Spit Tobacco Education Program. To improve health and health care 1998–1999.

[8] Gwet K (2008). Computing inter-rater reliability and its variance in the presence of high agreement. Br J Math Stat Psychol.

[9] Hoyle R, Stephenson M, Palmgreen P (2002). Reliability and validity of a brief measure of sensation seeking. Personal Individ Differ.

[10] Stephenson MT, Hoyle RH, Palgreen P, Slater M (2003). Brief measures of sensation seeking for screening and large-scale survey. Drug Alcohol Depend.

[11] StataCorp. (2019). Stata statistical software: release 16.

[12] Duke JC, Farrelly MC, Alexander TN (2018). Effect of a national tobacco public education campaign on youth’s risk perceptions and beliefs about smoking. Am J Health Promot.

[13] Farrelly MC, Healton CG, Davis KC (2002). Getting to the truth: evaluating national tobacco countermarketing campaigns. Am J Public Health.

[14] Farrelly MC, Davis KC, Duke J (2009). Sustaining ‘truth’: changes in youth tobacco attitudes and smoking intentions after 3 years of a national antismoking campaign. Health Educ Res.

[15] Sly DF, Heald GR, Ray S (2001). The Florida “truth” anti-tobacco media evaluation: design, first year results, and implications for planning future state media evaluations. Tob Control.

[16] Sharapova S, Reyes-Guzman C, Singh T (2020). Age of tobacco use initiation and association with current use and nicotine dependence among US middle and high school students, 2014–2016. Tob Control.

[17] Duke JC, MacMonegle AJ, Nonnemaker JM (2019). Impact of the real cost media campaign on youth smoking initiation. Am J Prev Med.

[18] Davis KC, Farrelly MC, Messeri P (2009). The impact of national smoking prevention campaigns on tobacco-related beliefs, intentions to smoke and smoking initiation: results from a longitudinal survey of youth in the United States. Int J Environ Res Public Health.

[19] Farrelly MC, Nonnemaker J, Davis KC (2009). The influence of the national truth campaign on smoking initiation. Am J Prev Med.

