# Supplementary material for: “The Real Cost” Smokeless campaign: changes in beliefs about smokeless tobacco among rural boys, a longitudinal randomized controlled field trial
Source: BMC Public Health. 2021 Dec 14;21:2282. doi: 10.1186/s12889-021-12356-6 (PMC8670032; doi:10.1186/s12889-021-12356-6)
Supplement: Supplementary file 1 — Additional file 1. [file 12889_2021_12356_MOESM1_ESM.docx]

**Supplement 1.** List of Explicit, Implicit, and Unrelated Beliefs Tested in “The Real Cost” Smokeless Evaluation

**Beliefs Explicitly Messaged in Campaign Advertisements**

- If I use smokeless tobacco, I will damage my body.
- If I use smokeless tobacco, I will be controlled by smokeless tobacco.
- If I use smokeless tobacco, I will develop cancer of the lip, mouth, tongue, or throat.
- If I use smokeless tobacco, I will be unable to stop when I want to.
- If I use smokeless tobacco, I will lose my teeth.
- If I use smokeless tobacco, I will shorten my life.
- If I use smokeless tobacco, I will miss out on things I enjoy doing.
- If I use smokeless tobacco, I will develop gum disease.
- If I use smokeless tobacco, I will develop red or white patches in the mouth.
- If I use smokeless tobacco, I will consume harmful chemicals.
- If I use smokeless tobacco, I will lose my jaw.
- Using smokeless tobacco can cause immediate damage to my body.

**Beliefs Implicitly Messaged in in Campaign Advertisements**

- If I use smokeless tobacco, I will be more attractive.
- If I use smokeless tobacco, I will fit in.
- If I use smokeless tobacco, I will gross out people I want to date.
- It is safe for me to use smokeless tobacco for only a year or two, as long as I quit after that.
- Using smokeless tobacco is a way to show others you’re not afraid to take risks.
- Using smokeless tobacco is a manly thing to do.

**Beliefs Unrelated to Campaign Advertisements**

- If I use smokeless tobacco, I will develop sexual and/or fertility problems.
- If I use smokeless tobacco, I will get sick more often.
- If I use smokeless tobacco, I will end up wasting money on smokeless tobacco.
- If I use smokeless tobacco, I will feel more relaxed.
- If I use smokeless tobacco occasionally, I will not become addicted.
- Using smokeless tobacco helps people relieve stress.
- Using smokeless tobacco is disgusting.
